# Mojabanchromanol Isolated from *Sargassum horneri* Attenuates Particulate Matter Induced Inflammatory Responses via Suppressing TLR2/4/7-MAPK Signaling in MLE-12 Cells

**DOI:** 10.3390/md18070355

**Published:** 2020-07-08

**Authors:** Kalahe Hewage Iresha Nadeeka Madushani Herath, Hyo Jin Kim, Jae-Hyuk Jang, Hyun-Soo Kim, Hyun Jung Kim, You-Jin Jeon, Youngheun Jee

**Affiliations:** 1 Department of Veterinary Medicine and Veterinary Medical Research Institute, Jeju National University, Jeju 63243, Korea; madushaniherath001@gmail.com; 2 Department of Food Bioengineering, Jeju National University, 102 JeJudaehakno, Jeju 63243, Korea; jyouding@gmail.com (H.J.K.); hyunjkim@jejunu.ac.kr (H.J.K.); 3 Natural Medicine Research Center, Korea Research Institute of Bioscience and Biotechnology, 30 Yeongudanji-ro, Ochang-eup, Cheongwon-gu, Cheongju-si, Chungcheongbuk-do 28116, Korea; jangjh@kribb.re.kr; 4 National Marine Biodiversity Institute of Korea, 75, Jangsan-ro 101-gil, Janghang-eup, Seocheon, Chungcheongnam-do 325-902, Korea; gustn783@mabik.re.kr; 5 Department of Marine Life Science, School of Marine Biomedical Sciences, Jeju National University, Jeju 690-756, Korea; youjin2014@gmail.com; 6 Interdisciplinary Graduate Program in Advanced Convergence Technology & Science, Jeju National University, Jeju 63243, Korea

**Keywords:** Mojabanchromanol, TLR2/4/7, MAPK, IL-1β, IL-6, *Sargassum horneri*

## Abstract

Chromanols from marine algae are studied for drug development due to its prominent bioactive properties, and mojabanchromanol (MC), a chromanol isolated from a brown algae *Sargassum horneri*, is found to possess anti-oxidant potential. In this study, we hypothesized MC may attenuate particulate matter (PM)-induced and reactive oxygen species (ROS)-mediated inflammatory responses in airways and tried to identify its potential and underlying mechanism against PM (majority <2.5 µm in diameter)-induced inflammatory responses in a lung type II alveolar epithelial cell line, MLE-12. MC attenuated PM-induced malondialdehyde (MDA), a lipid peroxidation end product, and 8-hydroxydeoxyguanosine (8-OHdG), the most representative DNA oxidative damage product, further validating MC’s potential in attenuating PM-induced oxidative stress. MC also suppressed PM-triggered TLR2/4/7 activation in MLE-12 cells. Moreover, MC reduced ROS-mediated phosphorylation of mitogen-activated protein kinase (MAPK) extracellular signal-regulated kinase 1/2 (Erk1/2) and c-Jun NH (2)-terminal kinase (JNK) that were also activated in PM exposed cells. MC further inhibited the secretion of pro-inflammatory cytokines (IL-6, IL-1β and IL-33) in MLE-12 cells exposed to PM. These results provide a clear evidence for MC’s potential in attenuating PM-triggered inflammatory responses in MLE-12 cells via repressing TLR2/4/7 and MAPK signaling. Therefore, MC can be developed as a therapeutic agent against PM induced airway inflammatory responses.

## 1. Introduction

Air pollution with particulate matter (PM) has become a worldwide concern due to its deleterious health effects, even leading to mortality, recently [1]. Epidemiological studies revealed a positive correlation between PM inhalation and increased risk of pulmonary diseases such as asthma, chronic obstructive pulmonary disease, cancer, etc. [2,3]. PM’s aerodynamic diameter and the chemical composition affect its biological influence on human health, but they can vary with the source, season, and location [4]. Ground measurements identified that a large proportion of PM consists of particles of aerodynamic diameter <2.5 µm, with major components like polycyclic aromatic hydrocarbons (PAH), heavy metals, metals, and organic chemicals [5]. Due to its small size, it can suspend in the air for a long time and can be easily inhaled and deposit in deep airways even in alveoli, causing more damages on alveolar epithelial cells than on other cells [6]. Because of this, alveolar type II epithelial cells (AECII) gained considerable attention due to its primary role in forming the alveolar barrier, surfactants, and repairing the damaged AECI [7]. 

Previous evidence reported that toxic components in PM are determinants of AECII injury [8]. In particular, PM’s components such as metals and PAH generate ROS and extensively damage lipids and DNAs [8,9]. PM is further known to increase the level of lipid peroxidation byproduct malondialdehyde (MDA) in A549 cells (AECII cell line) [10]. We previously revealed that PM enhances the level of 8-hydroxydeoxyguanosine (8-OHdG), the most representative product of DNA oxidative damage, in MLE-12 cells (AECII cell line). Previous studies also reported increased level of MDA and 8-OHdG in PM exposed mice, and that their increased level in body fluid can be used as biomarkers of oxidative stress in inflammatory diseases [11,12]. ROS and ROS-induced DNA damages are also identified to trigger pulmonary inflammation and fibrosis [13]. Furthermore, PM alone can activate toll like receptors (TLRs), mitogen-activated protein kinases (MAPKs), and nuclear factor (NF)-ҡB signaling pathways, leading to the activation of inflammatory responses probably via secreting pro-inflammatory cytokines (interleukin (IL)-1β, tumor necrosis factor (TNF)-α, IL-6, IL-33), and epithelial cell derived chemokines (IL-8, monocyte chemoattractant protein-1 (MCP-1)) [14]. These findings pinpoint that blocking oxidative stress mediated inflammatory responses might be beneficial in avoiding further exacerbation and occurrence of asthma like diseases due to PM. 

**Figure 1:**
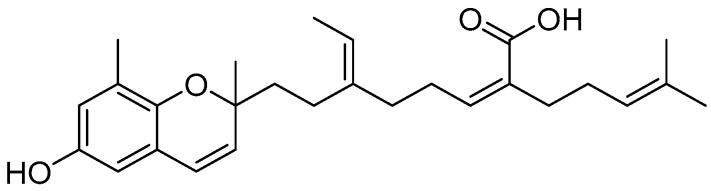
Chemical structure of mojabanchromanol.

Since current therapeutics such as steroids are ineffective against long-term management of inflammatory diseases because of a multitude of severe side effects, there are great interests in developing natural products as effective sources of novel drug candidates. *Sargassum horneri* is an edible brown algae and has been used as a medicinal herb to treat inflammatory diseases, heart diseases, and goiters in traditional Chinese medicine [15,16]. An array of phytochemicals found in *S. horneri* such as polyphenol, polysaccharides, and chromanols have been reported for promising anti-inflammatory, anti-tumor, and anti-oxidant properties [17,18]. Mojabanchromanol (MC) is a chromene of molecular weight 424.263 isolated from *S. horneri* (and later from *S. siliquastrum* by Jang et al.) (Figure 1) [18,19]. MC exhibited anti-oxidant effect with high DPPH radical elimination power (96.07%) and high thiobarbituric acid reactive substances (TBARS, 84.08% at 0.5 mg/mL) [18]. Moreover, we recently identified that MC exerts anti-allergy effect on bone marrow-derived mast cells [20]. We further disclosed that MC rich ethanol extract of *S. horneri* (SHE) attenuates the pathogenesis of PM-exacerbated asthma by attenuating airway hyper responsiveness, IgE secretion, mast cell activation, mucus hypersecretion, goblet cell hyperplasia via inhibiting Th2/Th17 responses [21]. 

Although MC’s anti-oxidant and anti-allergy effects are well defined, there’s no information on its role against inflammatory responses, and we explored its preventative effects and mechanisms against PM-induced inflammatory responses in this study. We examined systemic responses such as TLRs activation, downstream of MAPK signaling pathway, and the secretion of pro-inflammatory cytokines upon PM exposure and MC’s role against them. In particular, we used MLE-12, a frequently used AEC-II, as a model system to mimic the effects of deep penetrated PM-induced pulmonary inflammation in this study.

## 2. Results

### 2.1. Low Concentration MC Is Not Cytotoxic and Does Not Affect Cell Proliferation of MLE-12 Cells

**Figure 2:**
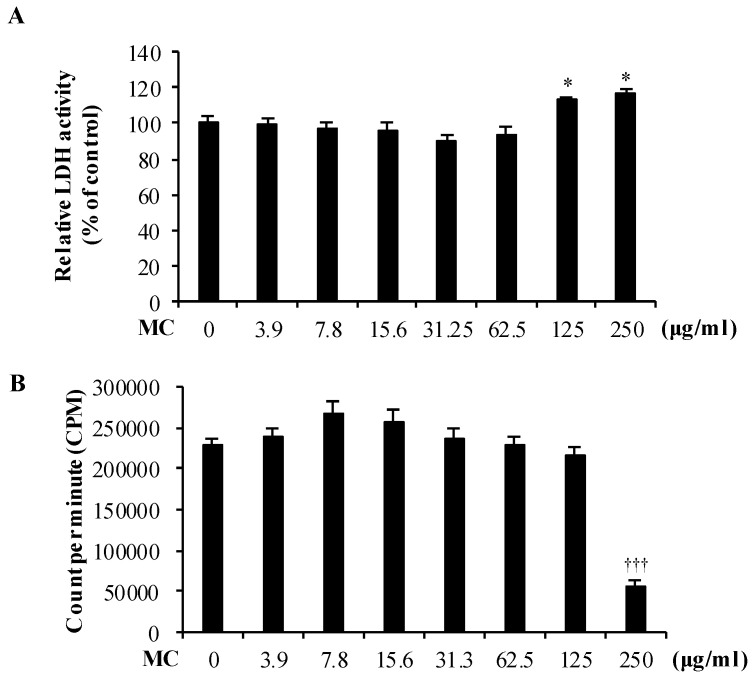
The effect of MC on (**A**) cytotoxicity and (**B**) proliferation of MLE-12 cells was evaluated. Data are represented as the mean ± S.E.M of at least three independent experiments (three replicates in each time). * (*p* < 0.05) represents significant difference against untreated control. **†††** (*p* < 0.0005) represents significant difference against PM only.

We first assessed the cytotoxicity of MC on MLE-12 cells using LDH assay. MC was not cytotoxic at concentrations 0–62.5 µg/mL, but became cytotoxic at higher concentrations (>125 µg/mL). The LDH release increased by 12.9% and 16.4% (both *p* < 0.05) at respective MC concentrations 125 and 250 µg/mL (Figure 2A). Similarly, low concentrations of MC (0–125 µg/mL) did not affect the proliferation of MLE-12 cells, but, at the highest concentration tested (250 µg/mL), MC exhibited significant reduction of cell proliferation (Figure 2B). These results suggested that MC at low concentrations (0–62.5 µg/mL) is not toxic and does not affect the proliferation of MLE-12 cells. Considering this, concentrations 31.3 and 62.5 µg/mL were selected as effective concentrations and used in later experiments. 

### 2.2. MC Attenuates PM-Triggered Cytotoxicity of MLE-12 Cells

**Figure 3:**
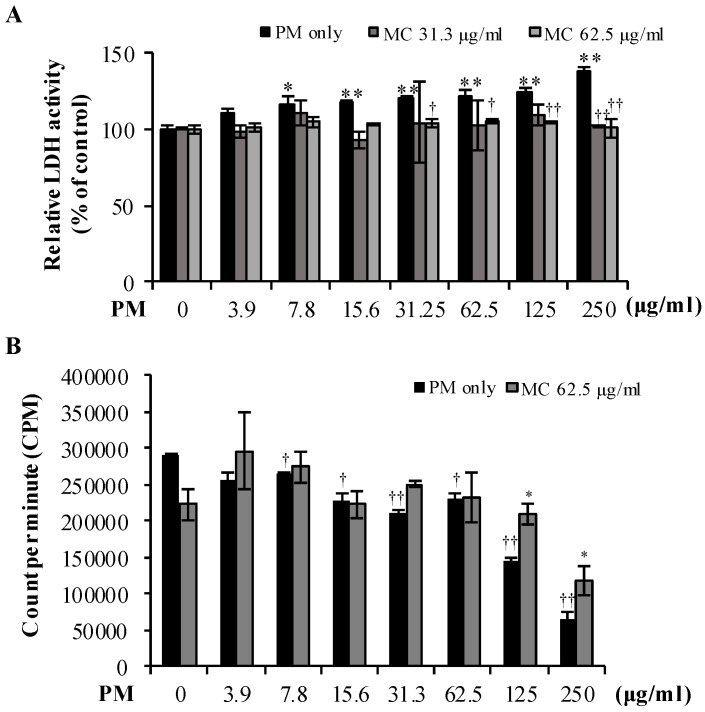
(**A**) Cytoprotective effect of MC on PM-exposed MLE-12 cells evaluated using LDH release. * (*p* < 0.05) and ** (*p* < 0.005) represent significant difference against untreated control. **†** (*p* < 0.05) and **††** (*p* < 0.005) represent significant difference against PM alone. (**B**) MC’s effect on the proliferation of PM-exposed MLE-12 cells measured using [^3^H] incorporation assay. **†** (*p* < 0.05) and **††** (*p* < 0.005) represent significant difference against untreated control. * (*p* < 0.05) represents significant difference against PM alone. Data are represented as the mean ± S.E.M of at least three independent experiments (three replicates in each time).

We then evaluated the cytotoxicity of PM on MLE-12 cells. As shown in Figure 3A (black bars), PM exerted cytotoxic effect even at a dose as low as 7.8 µg/mL (*p* < 0.05), and the cytotoxicity increased as the dose increased (by 1.2-fold at both 62.5 and 125 µg/mL (both *p* < 0.005), by 1.4-fold at 250 µg/mL (*p* < 0.005) compared to untreated control, respectively) in LDH assay. However, MC counterbalanced the PM-triggered LDH release and restored it to the level similar to that in untreated control although the reduction of LDH release was more pronounced at high concentrations of MC (62.5 µg/mL) and PM (31.25, 62.5 µg/mL (*p* < 0.05) and 125, 250 µg/mL (*p* < 0.005), respectively) (Figure 3A).

In parallel, we measured the proliferation of PM exposed cells using the ^3^H-thymidine incorporation assay. PM increasingly decreased the proliferation of MLE-12 cells at increasingly high concentrations (Figure 3B, black bars). But interestingly, MC treatments at 62.5 µg/mL could reverse the proliferation reduction in PM exposed MLE-12 cells by 1.4-fold at PM 125 µg/mL and by 1.8-fold at PM 250 µg/mL, respectively (both *p* < 0.05; Figure 3B). These results signify the cytoprotective effect of MC on PM exposed MLE-12 cells. Also, considering the successful cytotoxicity results of PM, we decided to use the concentration 125 µg/mL henceforth. 

### 2.3. MC Suppresses the Lipid Peroxidation in PM-Exposed MLE-12 Cells

**Figure 4:**
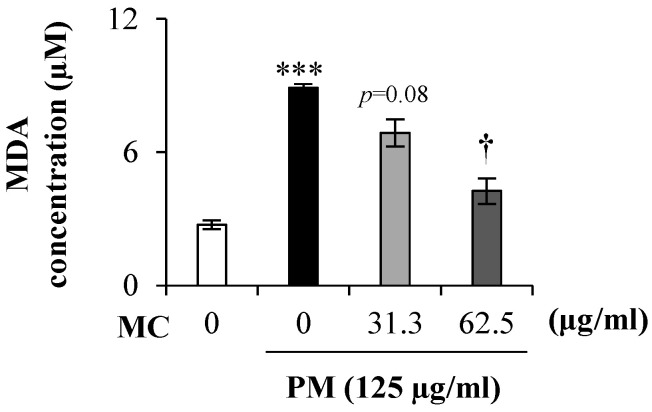
MC’s effect on the lipid peroxidation in PM-exposed MLE-12 cells as determined using MDA level. *** (*p* < 0.0005) represents significant difference against untreated control. **†** (*p* < 0.05) represents significant difference against PM alone. Data are represented as the mean ± S.E.M of at least three independent experiments (three replicates in each time).

It is known that PM triggers oxidative stress upon exposure, and prolonged oxidative stress leads to lipid peroxidation. To confirm this and to assess MC’s effect on it, we measured the level of lipid peroxidation product MDA in the culture supernatant of PM-exposed (125 µg/mL) MLE-12 cells. As expected, PM triggered the MDA level spike by 2.5-fold (*p* < 0.0005) compared to untreated control, but MC treatments dose dependently reduced the spiked MDA level (by 2.1-fold at 62.5 µg/mL (*p* < 0.05) in particular) in PM-exposed MLE-12 cells (Figure 4).

**Figure 5:**
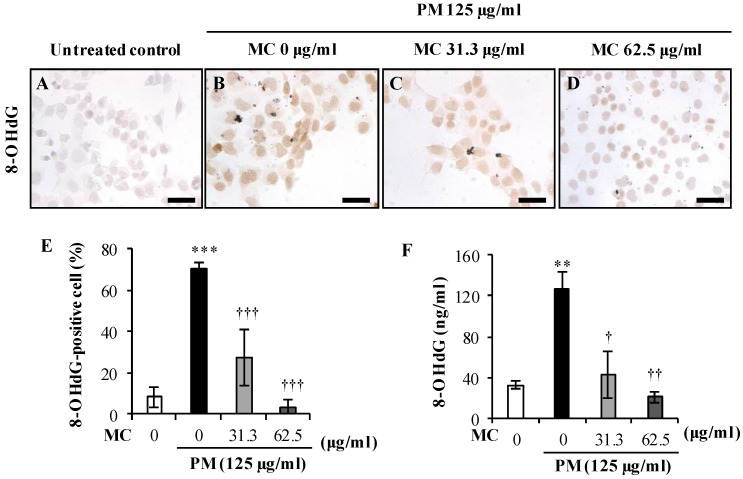
Effect of PM and MC on NF-κB activation in MLE-12 cells as determined by 8-OHdG positive cells. (**A**–**D**) Representative images of 8-OHdG positive cells taken at 3 h are shown. (**E**) The percentage of 8-OHdG positive cells determined using immunocytochemistry. (**F**) Level of 8-OHdG produced in PM-exposed MLR-12 cells. ** (*p* < 0.005), *** (*p* < 0.0005) represent significant difference against untreated control. **†** (*p* < 0.05), **††** (*p* < 0.005) and **†††** (*p* < 0.0005) represent significant difference against PM alone. Data are represented as the mean ± S.E.M of at least three independent experiments (three replicates in each time). Scale bars of **A**–**D** are 25 µm (×400 magnification).

In addition, we measured the expression of 8-OHdG, a marker of oxidative DNA damage, in MLE-12 cells using immunocytochemistry. As before, PM (Figure 5B,E) significantly increased the percentage of 8-OHdG expressing cells compared to untreated control (Figure 5A,E; *p* < 0.0005). However, MC dose dependently suppressed it in PM-exposed MLE-12 cells (by 2.6-fold at 31.3 μg/mL and by 20.0-fold at 62.5 μg/mL, respectively (both *p* < 0.0005)). When measured in the culture supernatant (Figure 5F), we observed 3.9-fold increase (*p* < 0.005) of 8-OHdG level in MLE-12 cells exposed to PM alone. However, in comparison to the PM alone group, we observed reduced accumulation of 8-OHdG in MC treated groups (by 3.0-fold at MC 31.3 μg/mL and by 6.0-fold at MC 62.5 μg/mL, respectively (both *p* < 0.05)). These results suggest MC attenuates lipid peroxidation and DNA damage due to PM-triggered oxidative stress.

### 2.4. MC Attenuates the PM-Triggered Activation of MAPK Pathway in MLE-12 Cells

**Figure 6:**
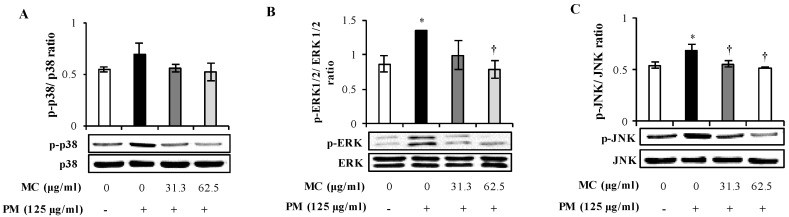
Effect of MC on PM-induced MAPK activation in MLE-12 cells. Densitometric analysis of the fold change of cytosolic (**A**) p-p38/p38, (**B**) p-ERK1/2/ERK1/2, and (**C**) p-JNK/JNK and their representative immunoblots at 3 h are shown. The intensity of each band was determined as a ratio to its corresponding loading in control band. * (*p* < 0.05) represents significant difference against untreated control, and **†** (*p* < 0.05) represents significant difference against PM alone. Data are represented as the mean ± S.E.M of at least three independent experiments (two replicates in each time).

PM-triggered inflammatory responses are known to be coordinated primarily through the activation of MAPK and NF-ҡB signaling pathways. However, we earlier identified that PM is linked to the activation of MAPK rather than NF-ҡB signaling in MLE-12 cells (Submitted, Herath et al., 2020). Therefore, we examined MC’s effect on PM-triggered MAPK activation in MLE-12 cells using western blot. As shown in Figure 6, PM alone triggered the activation of ERK and JNK by 1.6 and 1.2 folds (both *p* < 0.05) respectively compared to untreated control, whereas p38 was not affected. Moreover, we detected 46 kDa isoform of p-JNK in MLE-12 cells as observed similarly by Morales et al., 2014 [22] in A549 lung epithelial cells. Interestingly, however, MC dose dependently inhibited the phosphorylation of ERK (by 1.4-fold at 31.3 μg/mL and by 1.7-fold at 62.5 μg/mL, respectively (both *p* < 0.05)) and JNK (by 1.4-fold at 31.3 and 62.5 μg/mL, respectively (both *p* < 0.05)) to the level similar in untreated control, validating MC’s potential in suppressing PM-triggered MAPK signaling by repressing ERK and JNK. 

### 2.5. MC Suppresses the Secretion of Pro-inflammatory Cytokines in PM-Exposed MLE-12 Cells

**Figure 7:**
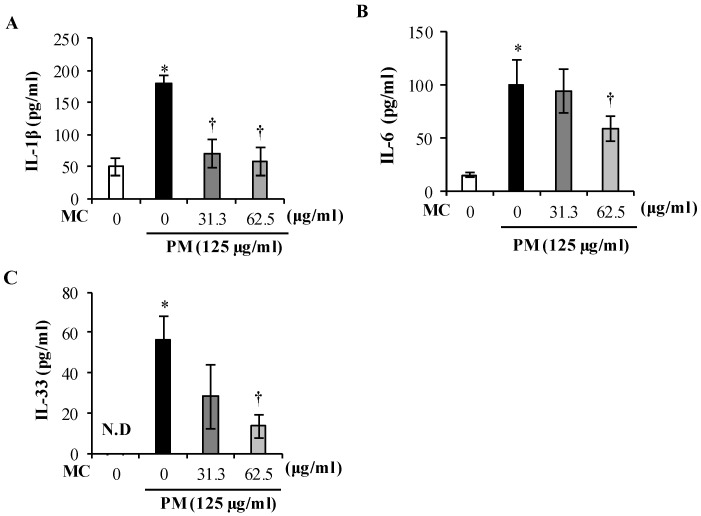
Effect of MC on PM-triggered secretion of pro-inflammatory cytokines (**A**) IL-1β, (**B**) IL-6, and (**C**) IL-33 in culture supernatants of MLE-12 at 3 h. Data are represented as the mean ± S.E.M of at least three independent experiments. * (*p* < 0.05) represents significant difference against untreated control, and **†** (*p* < 0.05) represents significant difference against PM alone.

The chemical and biological components in PM as well as the PM triggered oxidative stress are known to instigate alveolar epithelial cells to release a battery of pro-inflammatory cytokines including IL-1β, IL-6, and IL-33 [23]. So we analyzed the effect of MC on PM-triggered secretion of pro-inflammatory cytokines IL-1β, IL-6, and IL-33 in culture supernatants of PM exposed MLE-12 cells. As shown in Figure 7A, PM increased the secretion of IL-1β by 3.6-fold (*p* < 0.05), but the production of IL-1β was significantly suppressed by 2.5-fold and 3.1-fold with MC treatments of 31.3 and 62.5 µg/mL (*p* < 0.05), respectively, rendering them to the level in untreated control. We also observed significant upswing of pro-inflammatory cytokine IL-6 secretion (by 6.4-fold, *p* < 0.05) in PM exposed MLE-12 cells compared to untreated control, and MC decreased it gradually further with higher concentrations though it did not return to the level in untreated control at the highest concentrations tested (Figure 7B). In addition, IL-33 which was not detected in untreated control was dramatically increased by 56.4-fold (*p* < 0.05) when MLE-12 cells were exposed to PM (Figure 7C). But MC evidently reversed the production of IL-33 dose dependently (by 2.0-fold at 31.3 μg/mL and by 4.2-fold at 62.5 μg/mL (*p* < 0.05), respectively). These observations validate MC’s potential in attenuating PM-triggered secretion of pro-inflammatory cytokines IL-1β, IL-6, and IL-33 from alveolar epithelial cells and preventing PM-triggered inflammatory signals from spreading to tissues beyond the point of primary contact. 

### 2.6. MC Attenuates the PM-Triggered Activation of TLR2/4/7 in MLE-12 Cells

**Figure 8:**
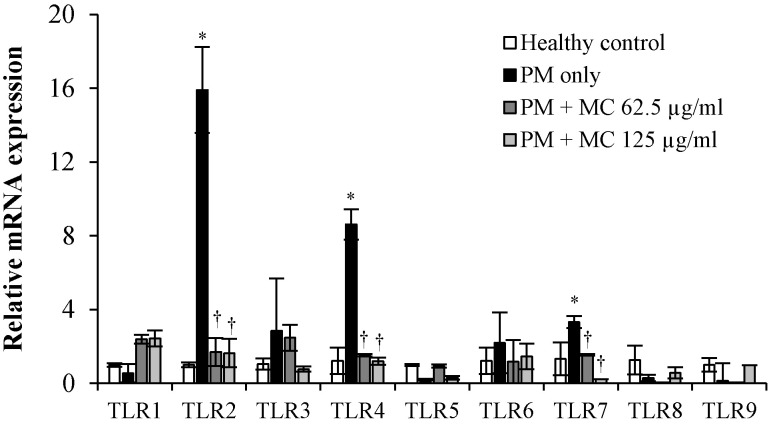
The effect MC on the PM-induced expression of TLRs on MEL-12 cells. Results of RT-PCR analysis of mRNA expression of TLR1-9 at 3 h are shown. * (*p* < 0.05) represents significant difference against untreated control, and **†** (*p* < 0.05) represents significant difference against PM alone. Data are represented as the mean ± S.E.M of at least three independent experiments.

We previously showed that various components in PM trigger the mRNA expression of TLR2/4/7 in MLE-12 cells (submitted, Herath et al., 2020). Considering the importance of TLRs in inflammatory reactions to allergens, we also investigated the role of MC on the regulation of mRNA expression of TLRs in MLE-12 cells (Figure 8). As expected, PM increased the mRNA expression of TLR2, TLR4, and TLR7 respectively by 16.0, 8.6 and 3.3 folds (all *p* < 0.05) in MLE-12 cells, and MC markedly attenuated their mRNA expression compared to PM only at both tested concentrations: TLR2 by 9.9-folds at both 31.3 and 62.5 µg/mL, TLR4 by 7.5-fold at 31.3 µg/mL and by 7.2-fold at 62.5 µg/mL, and TLR7 by 2.2-fold at 31.3 µg/mL and by 6.0-fold at 62.5 µg/mL, respectively, (all *p* < 0.05). These results clearly suggest that MC’s potential in attenuating PM-triggered inflammatory responses might be mediated via the attenuation of PM-triggered activation of TLR2/4/7 in MLE-12 cells.

## 3. Discussion

Seaweeds are rich in phytochemicals of immense medicinal value, and these phytochemicals have gained considerable attention in new drug development to treat inflammatory disorders such as allergy, etc. Chromanols are phenolic compounds with a ring of chromanols and aliphatic side-chains, and are naturally occurring anti-oxidants [24]. In this study, we investigated the protective effect and underlying mechanism of MC, a chromanol isolated from *S. horneri*, against PM-induced inflammatory responses in an AECII cell line MLE-12. To our knowledge, anti-inflammatory potential of MC is not reported so far. It is well recognized that inhaled PM plays a detrimental role, particularly in epithelial cells, by inducing oxidative stress and/or inflammatory responses [4,25]. In our recent study, we identified that PM induces ROS, secretes various pro-inflammatory mediators and cytokines, and produces inflammatory responses in MLE-12 cells. Therefore, attenuating these can be an efficient therapeutic strategy against PM-induced inflammatory responses in airways. 

Several studies identified the presence of environmentally persistent free radicals in PM’s surfaces. These free radicals such as ROS attack lipids and damage DNAs, resulting in the production of respective end products like MDA and 8-OHdG, which in turn can lead to cell death and lung injury when excessively prolonged or not handled promptly [26]. Moreover, metals on PM (Mg, Zn, Cd, L, Zn, Cu, Sr) are reported to elevate urinary MDA level in PM exposed human [27]. Several studies reported elevated formation of DNA oxidative damage biomarker 8-OHdG in lung epithelial cells, and asthmatic lungs in mice when exposed to PM [9,28]. In accordance with previous results on MC’s anti-oxidant potential, we also observe that MC attenuates the level of oxidative stress in PM-exposed MLE-12 cells in this study. The chromanol ring is also identified to scavenge free radicals and thereby attenuate oxidative stress recently [29]. 

An experimental study showed that inflammatory stimuli including transition metals and organic compounds can induce pulmonary inflammation in lungs exposed to inhaled PM via the activation of ERK, JNK, and p38 MAPK signaling and NF-ҡB pathways [30]. We previously identified that PM is linked to prominent MAPK activation in MLE-12 cells (Submitted, Herath et al., 2020). Interestingly, treating with MC attenuated the PM-activated ERK and JNK signaling pathways in MLE-12 cells. To the best of our knowledge, we are the first to report MC’s potential in attenuating PM-triggered activation of MAPK signaling pathway and alleviating the inflammatory damages due to PM. These observations indicate that MC protected alveolar epithelial cells from the progression of inflammatory responses induced by deep penetrated PM. Interestingly, other chromanols isolated from Sargassum sp. such as sargachromanol G, sargachromanol E were also shown to have strong inhibition of MAPK cascade members in 264.6 RAW cells when challenged by LPS [31,32].

Activated MAPK pathway, upon PM exposure, was identified to mediate the secretion of pro-inflammatory cytokines IL-1β, IL-6, and IL-33 in alveolar epithelial cells [33,34,35]. PM-induced IL-6 is essential for initiating inflammation, coagulation, and fibrin deposition in lung [36,37]. IL-6^−/−^ mice do not exhibit lung inflammation and injury even when exposed to PM, suggesting that IL-6 antibody treatment can attenuate pulmonary inflammation and injury [37]. Inhaled PM causes IL-1β secretion and further increases the thickness of conducting airways, mucus secretion, and lymphocyte accumulation in the airways, which can lead to COPD and asthma when persisted [38]. IL-33 is mainly secreted by the airway epithelium and evidenced to exacerbate the Th2-mediated inflammation via further promoting the secretion of IL-4, IL-5, and IL-13 in allergic patients with asthma [39]. Here, we found that MC can dose dependently attenuate the PM-induced secretion of pro-inflammatory cytokines (IL-1β, IL-6, and IL-33) in MLE-12 cells. Yoon et al. (2012) also identified anti-inflammatory potential of sargachromanol G, a chromanol isolated from *S. siliquastrum*, via attenuating the secretion of pro-inflammatory cytokines like tumor necrosis factor-α (TNF-α), IL-1β, and IL-6 in lipopolysaccharide induced 264.6 RAW cells [32]. Recently, it is reported that α-*tocopherol* in chromanol ring is responsible for the chromanol’s anti-inflammatory potential [40]. Our results attribute that MC probably exerts its anti-inflammatory effects through the suppression of MAPK signaling pathways.

The activation of TLRs by PM plays an important role in activating MAPK signaling cascade, and thereby triggering intracellular inflammatory signaling cascades. Therefore, TLRs activated upon PM exposure are useful candidate targets in attenuating PM-induced inflammatory responses. TLR2 and TLR4 are widely studied as potential biomarkers of PM exposed cells. A recent PM exposure study done with TLR2^−/−^ and TLR4^−/−^ BALB/c mice revealed that the absence of TLR2/4 significantly attenuates the progression of allergic inflammation [41]. Moreover, ROS are revealed to activate TLR2 and TLR4 in alveolar macrophages in lungs [42,43]. Apart from TLR2 and TLR4, we found that PM also activated TLR7 in MLE-12 cells. We assume that bacterial or viral nucleic acids present in PM might have activated the TLR7 in MLE-12 cells [44]. In our study, MC significantly attenuated the PM-triggered mRNA expression of TLR2, TLR4, and TLR7 in MLE-12 cells. Our study here apparently demonstrates for the first time that MC attenuates PM-induced activation of TLR2/4/7 in MLE-12 cells.

## 4. Materials and Methods

### 4.1. Reagents and Kits

LDH Cytotoxicity Detection Kit was purchased from Takara Bio Inc., (Kusatsu, Japan). HEPES buffer, hydrocortisone, β-estradiol, chloroform, and β-actin were purchased from Sigma-Aldrich (Saint Louis, MO, USA). Insulin-Transferrin-Selenium, l-glutamine, fetal bovine serum, Dulbecco’s modified eagle’s medium/nutrient mixture F-12 (DMEM/F12), and Trizol were purchased from Gibco Life Technologies (Grand Island, NY, USA). ^3^H-thymidine was purchased from Amersham Life Science (Arlington Heights, IL, USA). Primary antibodies for p38, phospho p38, ERK1/2, pERK1/2 were purchased from Cell Signaling Technology (Danvers, MA, USA), and those for JNK, pJNK were purchased from Santa Cruz Biotechnology (Dallas, TX, USA). Westzol was purchased from iNtRON Biotechnology (Sungnam, Korea). cDNA synthesis kit was purchased from Promega (St Louis, MO, USA). Power SYBER Green PCR Master Mix was purchased from Applied Biosystems (Foster City, CA, USA). Mouse IL-1β, IL-6 ELISA kits were purchased from BioLegend (San Diego, CA, USA). Mouse IL-33 ELISA kit was purchased from Invitrogen (Thermo Fisher Scientific, Waltham, MA, USA). Ethanol was purchased from OCI Chemicals (Seoul, Korea).

### 4.2. Particulate Matter

The environment certified material, No.28 urban aerosol purchased from Japanese National Institute for Environmental Studies was used in this study. We previously analyzed its size distribution and revealed that the majority of the PM particles are <2.5 µm of aero-dynamic diameter [45]. It contains heavy materials such as Ba (874 ± 65 mg/kg), Pb (403 ± 32 mg/kg), Sr (469 ± 16 mg/kg), and polycyclic aromatic hydrocarbons (seller’s website, https://www.nies.go.jp/labo/crm-e/aerosol.html).

### 4.3. Preparation of Seaweed and Isolation of MC

The brown algae *S. horneri* was collected from coasts of Jeju island, South Korea, and seaweed extracts were prepared as previously described [46]. In brief, washed algae was dried at 50 °C, and ground and passed through a 40–50 mesh. Obtained powder (100 g) was dissolved in ethanol (2 l) for 12 h, mixed well with white clay (2 h), and centrifuged (10,000 g, at RT). The separated supernatant was concentrated to 20% (solid basis) and treated with 95% ethanol to obtain a pure sample (SHE). 

We further purified the SHE and isolated its active component MC as described in [20]. Briefly, SHE was partitioned upon polarity using n-hexane (SHEH) and ethyl acetate. Using the ODS open column chromatography and HPLC analysis, we identified the active component as MC (UV absorption at 225 nm). Its structure is illustrated in detail elsewhere [20].

### 4.4. Cell Culture

For experiments, mouse alveolar type II epithelial cell line MLE-12 (American Type Culture Collection, Manassas, VA, USA) was used. Cells were cultured in DMEM/F12 medium containing 10 mM HEPES buffer, 10 nM hydrocortisone, 10 nM β-estradiol, 1% Insulin-Transferrin-Selenium, 2 mM l-glutamine, and 2% fetal bovine serum in an incubator (at 37 °C, 5% CO_2_) under proper humidity. Cells were subcultured at about 80% confluency, and the medium was changed at every 2 days. Viable cells (>90%) were seeded for experiments and incubated for 12 h to adhere to plates before treatments with PM or MC.

### 4.5. LDH Release Assay

Lactate dehydrogenase (LDH) release assay was performed to determine the cytotoxicity of PM and MC on MLE-12 cells. MLE-12 cells (1 × 10^3^ cells/well) seeded in triplicates on 96-well plates (Nunc) were exposed to either varying concentrations (0, 3.9, 7.8, 15.6, 31.3, 62.5, 125, and 250 µg/mL) of MC or PM alone or varying concentrations of PM together with selected concentrations of MC (0, 31.3, and 62.5 µg/mL) for 3 h. LDH release from damaged cells in the culture supernatant was determined at 490 nm using Multi-Mode Microplate Reader (Bio-Health Materials Core-Facility, Jeju National University). The LDH release in untreated control was determined as 100%, and experiment data are reported as the percentage of LDH release compared to that in untreated control.

### 4.6. ^3^H-thymidine Incorporation Assay

The modulation of cell proliferation by MC and/or PM was measured using ^3^H-thymidine incorporation assay as previously described [47]. MLE-12 cells (1 × 10^4^ cells/well) seeded in triplicates on 96-well plates (Nunc) were exposed to either varying concentrations (0, 3.9, 7.8, 15.6, 31.3, 62.5, 125, and 250 µg/mL) of MC or PM alone or varying concentrations of PM together with selected concentrations of MC (0, 31.3, and 62.5 µg/mL) for 54 h. After adding 1 μCi of ^3^H-thymidine (42 Ci/mmol specific activity), cells were incubated for 18 h, and then harvested on glass fiber filters to determine the amount of thymidine incorporated into DNA using a liquid scintillation spectrometer (Wallac Micro Beta^®^ TriLux, Perkin Elmer, Waltham, MA, USA).

### 4.7. Immunocytochemistry Analysis for 8-OHdG

MLE-12 cells (8 × 10^4^ cells/well, in triplicates) were seeded on coated coverslips in 12 well plates and treated with varying concentrations of MC (0, 31.3, and 62.5 µg/mL) with PM (0, 125 µg/mL). After 3 h incubation, cells were washed in ice cold PBS and fixed in 4% paraformaldehyde (in PBS, pH 7.4). Cells were then incubated with the primary antibody 8-OHdG (1:500) for 1 h at room temperature followed by incubation with the secondary biotinylated anti-rabbit IgG (for 30 min at RT). After washing, we used HRP-labeled Vectastain Elite ABC kits (Vector, Peterborough, UK) to perform the avidin-biotin peroxidase complex binding reaction. Immunoreactivity is visualized using 3,3’-diaminbenzidine (DAB, Vector, Peterborough, UK) as the substrate and counterstained with hematoxylin. Representative images were taken under an Olympus DP-72 (Olympus, Tokyo, Japan) microscope, and the percentage of 8-OHdG positive cells was quantified using ImageJ software (v1.46, National Institutes of Health, Bethesda, MD, USA).

### 4.8. ELISA

We performed ELISA analysis to assess the level of lipid peroxidation products (MDA, 8-OHdG-acethycholinesterase conjugate (DNA/RNA oxidative damage tracer)) and pro-inflammatory cytokines (IL-1β, IL-6, and IL-33) in the culture supernatant of PM-exposed MLE-12 cells. MLE-12 cells were treated with varying concentrations of MC (0, 31.3 and 62.5 µg/mL) with or without PM (0, 125 µg/mL) and allowed for incubation. After 3h, the culture supernatant was collected and analyzed for the level of MDA, 8-OHdG, IL-1β, IL-6, and IL-33 using respective ELISA kits according to manufacturers’ recommendations.

### 4.9. Western Blot Analysis for MAPK Signaling Pathway Activation

MLE-12 cells (1 × 10^6^ cells/dish) were treated with varying concentrations of MC (0, 31.3 and 62.5 µg/mL) with or without PM (0, 125 µg/mL) and allowed for 3h for incubation. Cells were then harvested, and cytosolic proteins of MLE-12 cells were extracted using the NE-PER^®^ Nuclear and Cytoplasmic extraction kits. Equal amount of protein lysates (40 μg) were separated by electrophoresis in 10% SDS-polyacrylamide gels. After being transferred onto a nitrocellulose membrane (100 V for 120 min), membranes were incubated with 1% skim milk (45 min, RT). Blots were separately incubated with primary antibodies of p-ERK1/2, ERK1/2, p-p38 MAPK, p38 MAPK, p-JNK, JNK, and β-actin (for 1 h at RT). After washing in TBST, the membranes were incubated with secondary goat anti-rabbit IgG-HRP or goat anti-mouse IgG-HRP (45 min, at RT). Specific signals were detected using Westzol ECL solutions, and densitometry analysis was performed using ImageJ software (v1.46, National Institutes of Health, Bethesda, MD, USA).

### 4.10. RNA Isolation, cDNA Preparation, and qPCR Analysis

MLE-12 cells were treated with MC (0, 31.3 and 62.5 µg/mL) with or without PM (0, 125 µg/mL) for 3 h. After washing in ice cold DPBS, cells were homogenized in Trizol regent, and RNA was isolated as described previously [21]. Single strand cDNAs were synthesized using promega A3500 cDNA synthesis kits, and all qPCR assays were performed on a StepOnePlus realtime PCR system (Applied Biosystems, Foster City, CA, USA) using 20 µL of reaction volumes with Power SYBER Green PCR Master Mix (Applied Biosystems, Foster City, CA, USA) in triplicates. We quantified the relative mRNA expression using 2^−ΔΔCT^ method, and relative expression of target genes was finally normalized to the endogenous control GAPDH. Primers used are indicated in Table 1.

### 4.11. Statistical Analysis

One-way analysis of variance (ANOVA) followed by Tukey’s multiple comparison was performed to compare values among multiple groups. Between two groups, Student’s *t*-test was performed. *p* value < 0.05 was considered statistically significant.

## 5. Conclusions

This is the first report to demonstrate MC’s anti-inflammatory potential in PM-exposed MLE-12 cells. Our results provide a clear evidence for the potential of MC from *S. horneri* to attenuate PM induced inflammatory responses in an alveolar epithelial cell line MLE-12. MC attenuated DNA damages and lipid peroxidation mediated through PM induced ROS. Moreover, MC exhibited its anti-inflammatory potential via suppressing the TLR2/4/7-mediated MAPK signaling cascade and attenuating PM-triggered secretion of pro-inflammatory cytokines (IL-1β, L-6 and IL-33). Therefore, we conclude that MC from *S. horneri* can be a potential cytoprotective agent against PM induced airway inflammatory responses.

## Figures and Tables

**Table 1:** Mouse primer sequences for quantitative PCR in MLE-12 cells and lung.

Gene	Sequence	
	Forward (5′-3′)	Reverse (5′-3′)
TLR1	GTT GTC ACT GAT GTC TTC AGC	CTG TAC CTT AGA GAA TTC TG
TLR2	CAG CTTA AAG GGC GGG TCA GAG	TGG AGA CGC CAG CTC TGG CTCA
TLR3	GAA GCA GGC GTC CTT GGA CTT	TGT GCT GAA TTC CGA GAT CCA
TLR4	AGT GGG TCA AGG AAC AGA AGC A	CTT TAC CAG CTC ATT TCT CAC C
TLR5	GAA TTC CTT AAG CGA CGT AA	GAG AAG ATA AAG CCG TGC GA
TLR6	AGT GCT GCC AAG TTC CGA CA	AGC AAA CAC CGA GTA TAG CG
TLR7	CCT GTT CTA CTG GGG TCC AA	GCC TCA AGG CTC AGA AGA TG
TLR8	GGC ACA ACT CCC TTG TGA TT	CAT TTG GGT GCT GTT GTT TG
TLR9	CCA GAC GCT CTT CGA GAA CC	GTT ATA GAA GTG GCG GTT GT
GAPDH	AAC GAC CCC TTC ATT GAC C	TCA GAT GCC TGC TTC ACC C

TLR: Toll like receptor; GAPDH: Glyceraldehyde 3-phosphate dehydrogenase.
